# Severe community-acquired pneumonia: Characteristics and prognostic factors in ventilated and non-ventilated patients

**DOI:** 10.1371/journal.pone.0191721

**Published:** 2018-01-25

**Authors:** Miquel Ferrer, Chiara Travierso, Catia Cilloniz, Albert Gabarrus, Otavio T. Ranzani, Eva Polverino, Adamantia Liapikou, Francesco Blasi, Antoni Torres

**Affiliations:** 1 Department of Pneumology, Respiratory Institute, Hospital Clinic, Institut d'Investigacions Biomèdiques August Pi i Sunyer (IDIBAPS), University of Barcelona, Barcelona, Spain; 2 Centro de Investigaciones biomedicas En Red-Enfermedades Respiratorias (CibeRes CB06/06/0028)-ISCIII, Barcelona, Spain; 3 Dipartimento di Fisiopatologia Medico-Chirurgica e dei Trapianti, Fondazione IRCCS Ca’ Granda-Ospedale Maggiore Policlinico, Università degli Studi di Milano, Milan, Italy; 4 Pulmonary Division, Heart Institute, Medical School, University of São Paulo, São Paulo, Brazil; 5 6^th^ Department of Respiratory Medicine, Sotiria Chest Diseases Hospital, Athens, Greece; National Yang-Ming University, TAIWAN

## Abstract

**Background:**

Patients with severe community-acquired pneumonia (SCAP) and life-threatening acute respiratory failure may require invasive mechanical ventilation (IMV). Since use of IMV is often associated with significant morbidity and mortality, we assessed whether patients invasively ventilated would represent a target population for interventions aimed at reducing mortality of SCAP.

**Methods:**

We prospectively recruited consecutive patients with SCAP for 12 years. We assessed the characteristics and outcomes of patients invasively ventilated at presentation of pneumonia, compared with those without IMV, and determined the influence of risks factors on mortality with a multivariate weighted logistic regression using a propensity score.

**Results:**

Among 3,719 patients hospitalized with CAP, 664 (18%) had criteria for SCAP, and 154 (23%) received IMV at presentation of pneumonia; 198 (30%) presented with septic shock. In 370 (56%) cases SCAP was diagnosed based solely on the presence of 3 or more IDSA/ATS minor criteria. *Streptococcus pneumoniae* was the main pathogen in both groups. The 30-day mortality was higher in the IMV, compared to non-intubated patients (51, 33%, *vs*. 94, 18% respectively, p<0·001), and higher than that predicted by APACHE-II score (26%). IMV independently predicted 30-day mortality in multivariate analysis (adjusted odds-ratio 3·54, 95% confidence interval 1·45–8·37, p = 0·006). Other independent predictors of mortality were septic shock, worse hypoxemia and increased serum potassium.

**Conclusion:**

Invasive mechanical ventilation independently predicted 30-day mortality in patients with SCAP. Patients invasively ventilated should be considered a different population with higher mortality for future clinical trials on new interventions addressed to improve mortality of SCAP.

## Introduction

Community-acquired pneumonia (CAP) is a significant cause of morbidity and mortality [1]. The definition of severe CAP (SCAP) is not univocal and this classification includes a heterogeneous group of patients. The criteria currently used to define SCAP in the guidelines are based on the presence of severe acute respiratory failure (ARF) needing invasive mechanical ventilation (IMV) and/or septic shock with organ system dysfunction [1,2]. Alternatively, several minor criteria requiring a high intensity monitoring and treatment have been proposed [1].

Severe CAP is associated with significant mortality, and despite effective antibiotic therapy, 16% to 36% patients may die within a short period of time [3–5]. Therefore, efforts to improve mortality of SCAP should be directed to select populations of patients at high risk of mortality.

Patients with SCAP and life-threatening ARF may require IMV [6]. However, the use of IMV is associated with multiple complications [7,8] and a high mortality [9]. The need for IMV may also be a marker of more severe acute disease regardless the use of this life-support measure. However, no prospective studies have comprehensively assessed the impact of IMV in consecutive series of patients with SCAP.

We hypothesized that IMV in patients with SCAP would result in worse outcomes regardless of their initial clinical severity. The aim of this study was therefore to identify a population of patients with SCAP characterized by a high mortality that could benefit from future clinical trials on treatments aimed at reducing mortality. Since IMV is a major determinant of CAP severity, and IMV is associated with higher mortality in patients with SCAP, we divided the population according to the use for IMV or not. Furthermore, we studied the risk factors for mortality, including invasive ventilatory support, in this critically ill population.

## Methods

### Patients

A prospective observational study was conducted at Hospital Clinic of Barcelona. All consecutive cases of CAP admitted from the Emergency Department between January-2000 and December-2011 were registered, and we selected all cases with SCAP. For publication purposes, the study was approved by the Ethics Committee of our institution (*Comité Ètic d’Investigació Clínica*, register: 2009/5451). Written informed consent was waived because of the non-interventional design.

Pneumonia was defined as a new pulmonary infiltrate on the admission chest radiograph, and symptoms and signs of lower respiratory tract infection. The exclusion criteria were: a) severe immunosuppression (human immunodeficiency virus infection, active solid or hematological neoplasm treated with chemotherapy, oral corticosteroid treatment with 20 mg or more prednisone-equivalent per day for at least two weeks, and other immunosuppressive drugs); b) active tuberculosis; c) a confirmed alternative diagnosis; and d) criteria of health-care associated pneumonia [10].

Severe CAP was defined according to the 2007 Infectious Disease Society of America/American Thoracic Society guidelines [1]. Patients presenting within the first 48 hours of hospital admission at least one major criteria, either septic shock or use of IMV or, in absence of major criteria, patients with at least three minor criteria, as described in Table 1, were selected for the present study. Presentation of these severity criteria after this period of time was considered clinical worsening. Because blood urea nitrogen level is not systematically determined in our hospital, we accepted, in its place, serum creatinine level >1.5 mg/dL [3,11].

**Table 1:** Frequency of severity criteria in the study population at presentation of pneumonia.

Severity criteria	n = 664
**Major criteria**	
Use of invasive mechanical ventilation	154 (23)
Septic shock	198 (30)
**Minor criteria**	
PaO_2_/FiO_2_ ≤250 *	392 (59)
Respiratory rate ≥30 breaths/min *	373 (56)
Creatinine level >1·5 mg/dL	357 (54)
Confusion/disorientation	318 (48)
Multilobar radiologic infiltrates	296 (45)
Hypotension (not meeting septic shock criteria)	101 (15)
Core temperature <36°C	72 (11)
White blood cell counts <4,000 cells/mm^3^	47 (7)
Platelet count <100,000 cells/mm^3^	26 (4)

Results are given as n (%).

* The use of non-invasive mechanical ventilation can substitute for respiratory rate ≥30 breaths/min or PaO_2_/FiO_2_ ≤250 [1].

The decisions to initiate IMV were taken by the attending physicians, based on the presence of any of the following intubation criteria: respiratory or cardiac arrest, respiratory pauses with loss of alertness or gasping for air, severely impaired consciousness, major agitation inadequately controlled by sedation, signs of exhaustion, massive aspiration, inability to manage respiratory secretions appropriately, and hemodynamic instability without response to fluids and vasoactive agents [12]. In addition, patients were also intubated in case of subsequent worsening of gas exchange or respiratory distress despite supportive measures.

### Data collection

The following parameters were recorded at admission: age, sex, current or former smoking (>10 pack-years), current or former alcohol (>80 g/day for at least one year before presentation) and drug consumption, co-morbidities, antibiotic treatment within 30 days before hospital admission, previous treatment with inhaled and systemic corticosteroids, clinical parameters, arterial blood gases, chest radiograph findings, including pleural effusion, laboratory parameters, adequacy of empiric antibiotic therapy, use of IMV, other clinical events (septic shock, acute renal failure). Admission to intensive care units (ICU), which included intermediate care units, the length of stay, and 30-day mortality were also noted. We also calculated the Acute Physiology And Chronic Health Evaluation (APACHE)-II score [13], the Pneumonia Severity Index (PSI) [14], and the CURB-65 (Confusion, elevated blood Urea nitrogen, Respiratory rate and Blood pressure plus age ≥65 years) score [15,16] at admission.

### Microbiologic evaluation

Sputum and two blood samples were obtained for bacterial culture before start of antibiotic therapy in the Emergency Department. Nasopharyngeal swabs for respiratory virus detection and urine samples for *Streptococcus pneumoniae* and *Legionella pneumophila* antigen detection were obtained within 24 hours after hospital admission. Pleural puncture, tracheobronchial aspirates and bronchoalveolar lavage fluid, when available, were collected for Gram and Ziehl–Nielsen stains and cultured for bacterial, fungal and mycobacterial pathogens. Blood samples for serology of atypical pathogens and respiratory virus was performed at admission and within the third and sixth weeks thereafter. Additional details and the criteria for etiologic diagnosis have been extensively described [17].

### Statistical analysis

We showed n (%) for categorical variables and mean±SD for continuous variables. Categorical variables were compared with the chi-square test or the Fisher exact test. Continuous variables were compared between 2 groups using the t-test or the analysis of variance (ANOVA) was used when comparing more than 2 groups. The ICU and hospital stay are shown as median (interquartile range), and were compared with the Mann-whitney non-parametric test due to the non-normally distributed values.

In addition to compare the characteristics and outcomes of patients with and without IMV, we distinguished those who met the major criteria from those who met the minor criteria only, according to IDSA/ATS 2007 definition [1].

Generalized linear model (GLM) analyses [18] were performed to determine the influence of the risks factors on 30-day mortality. Models were defined using a binomial probability distribution and a logit link function, using inverse probability of treatment weights (IPTWs) [19] to account for biases due to observed confounders. In a first step, each risk factor (age, smoking and alcohol consumption, co-morbidities, confusion/disorientation, multilobar infiltration, APACHE-II, PSI risk class, CURB-65, laboratory and blood gas variables, adequacy of empiric antibiotic therapy, shock, and use of IMV) was tested individually. In a second step, a propensity score (PS) for patients with IMV were developed. The PS was determined, irrespective of the outcome, through a multivariate logistic regression to predict the influence of 16 predetermined variables on the use of IMV. Variables were chosen for inclusion in the PS calculation according to the methods of Brookhart et al [20] and included variables associated with IMV use and outcome (age, gender, previous antibiotics, smoking and alcohol consumption, chronic respiratory, cardiovascular, neurological, renal, and liver disease, diabetes mellitus, APACHE-II, multilobar infiltration, pleural effusion, acute renal failure, and adequacy of empiric antibiotic therapy). IPTW used the PS to form a weight. The weights were finally incorporated in the multivariate weighted logistic regression model to predict 30-day mortality, including all risk factors which showed an association in the univariate analyses (p<0·10), and calculated in a stepwise backward elimination procedure, dropping non-significant variables until no further improvement of the Akaike’s information criterion was achieved [21]. The odds-ratio (OR) and 95% confidence intervals (CI) were calculated. Variables highly correlated were excluded from multivariate analyses. Goodness-of-fit information was given for the Pearson chi-square test to assess the overall fit of the model. The area under the receiver operating characteristic (ROC) curve of the multivariate model to predict 30-day mortality was calculated. All analyses were performed using the Observed Cases approach.

The level of significance was set at 0·05 (two-tailed). All analyses were performed with IBM SPSS Statistics 20.0 (Armonk, New York).

## Results

### Patients’ characteristics

**Fig 1:**
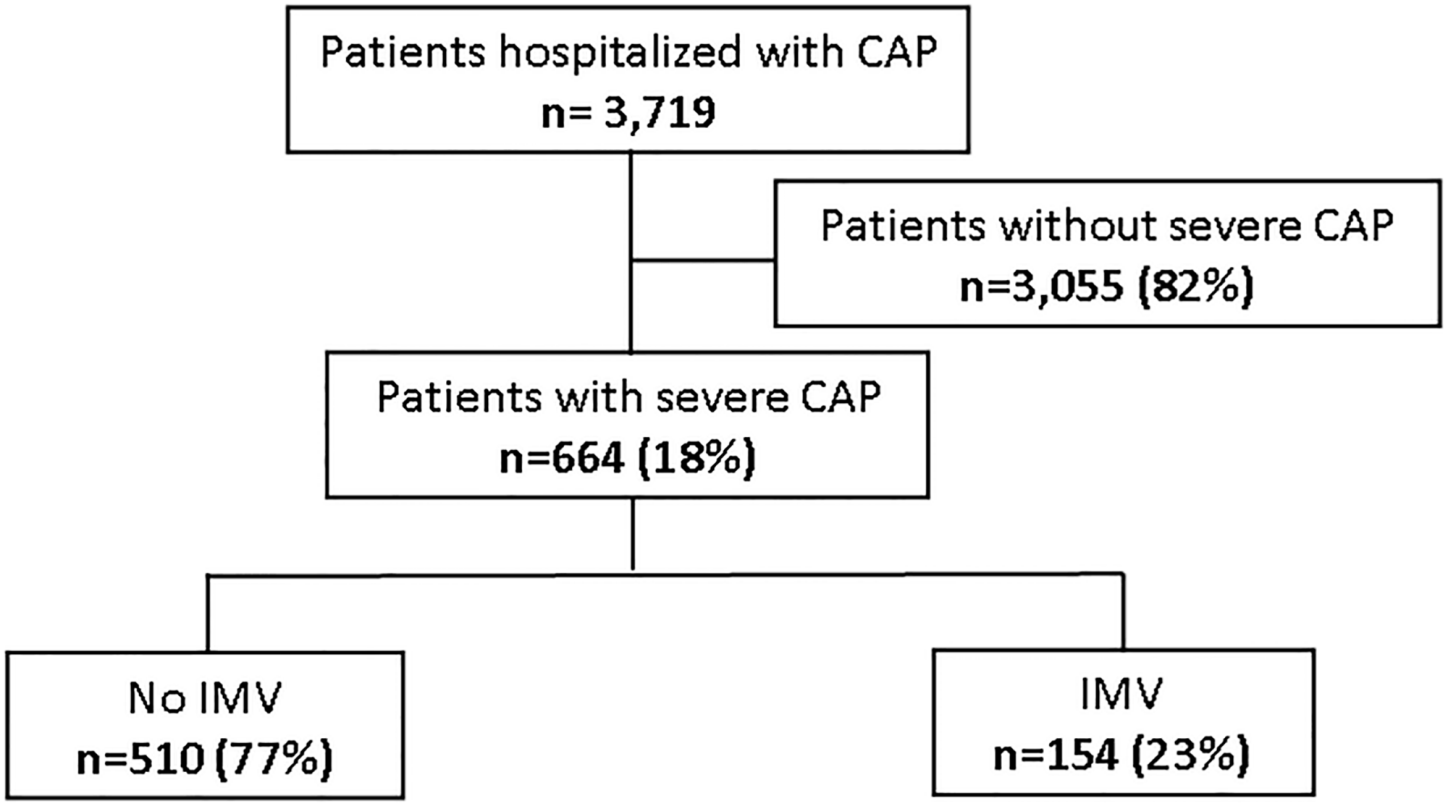
Flow diagram of the study population.

Among 3,719 patients with CAP diagnosis during the study period, 664 (18%) had criteria for SCAP; of those, 154 (23%) required IMV during the current hospital admission (Fig 1). Ninety-four (18%) patients without IMV had received non-invasive ventilation (NIV). The diagnosis of SCAP was based on the presence of major severity criteria in 294 (44%) cases; 154 patients were invasively ventilated and 198 had septic shock, with 58 having both major criteria. In 370 (56%) cases the diagnosis of SCAP was based solely on the presence of 3 or more minor criteria. The frequency of severity criteria in our population is shown in Table 1.

Patients from the IMV group were younger, had received less frequently previous antibiotics and influenza vaccine, at hospital admission they had higher heart rate and diastolic blood pressure, worse baseline oxygenation, higher arterial CO_2_ tension (PaCO_2_), lower arterial pH and CURB-65 score, less frequently acute renal failure, and a higher rate of pleural effusion and septic shock, with a trend to less frequent previous treatment with inhaled corticosteroids, and lower white blood cell count and hematocrite, compared to patients without IMV (Tables 2 and 3).

**Table 2:** General characteristics of the study population.

Variable	No IMV n = 510	IMV n = 154	p-value
Age (years)	72±16	66±16	**<0·001**
Sex (male)	337 (66)	103 (67)	0·85
Current or former smoking	308 (61)	91 (62)	0·91
Current or former alcohol abuse	115 (23)	42 (29)	0·14
Intravenous drug abuse	2 (0·4)	1 (1)	0·66
Previous antibiotics	114 (24)	20 (15)	**0·033**
Influenza vaccine	202 (49)	30 (36)	**0·030**
Pneumococcal vaccine	61 (15)	12 (14)	0·81
Previous inhaled corticosteroids	107 (21)	21 (14)	0·060
Previous systemic corticosteroids	12 (3)	6 (5)	0·21
Co-morbidities:			
Chronic respiratory disease *	217 (43)	63 (41)	0·71
Chronic cardiovascular disease ^±^	116 (23)	38 (25)	0·59
Diabetes mellitus	116 (24)	34 (23)	0·90
Chronic neurological disease	125 (25)	33 (22)	0·49
Chronic renal disease	62 (12)	16 (11)	0·59
Chronic liver disease	26 (5)	12 (8)	0·21

Results are given as n (%) or mean±SD. Percentages calculated on non-missing data.

* Chronic respiratory disease includes chronic obstructive pulmonary disease, asthma, bronchiectasis, and *sequelae* of pulmonary tuberculosis.

^±^ Chronic cardiovascular disease includes coronary artery disease, hypertensive or valvular heart diseases, and dilated myocardial disease of any cause. IMV = invasive mechanical ventilation; SD = standard deviation.

**Table 3:** Characteristics of pneumonia at hospital admission.

Variable	No IMV n = 510	IMV n = 154	p-value
Vital signs at hospital admission:			
Respiratory rate (breaths/min)	32±8	31±10	0·32
Heart rate (beats/min)	101±21	111±23	**<0·001**
Systolic blood pressure (mmHg)	123±32	128±36	0·090
Diastolic blood pressure (mmHg)	68±16	73±19	**0·006**
Laboratory data at hospital admission:			
Creatinine (mg/dL)	1·7±1·0	1·7±1·1	0·81
C-reactive protein (mg/dL)	21±13	22±14	0·59
White blood cells (10^9^ cell/L)	15·0±9·3	13·5±7·5	0·066
Hematocrite (%)	40±6	38±8	0·055
Platelets (10^9^ cell/L)	241±103	265±155	0·16
PaO_2_/FiO_2_ (mmHg)	236±66	213±84	**0·005**
PaCO_2_ (mmHg)	37±13	43±17	**<0·001**
Arterial pH	7·42±0·09	7·36±0·13	**<0·001**
Serum Na^+^ (mEq/L)	136±6	135±6	0·20
Serum K^+^ (mEq/L)	4·1±0·8	4·0±0·8	0·11
Severity variables at hospital admission:			
APACHE-II	16±5	17±6	0·091
PSI risk class IV-V	424 (83)	125 (81)	0·57
CURB-65 risk score 3–5	322 (63)	76 (49)	**0·002**
Confusion/disorientation	245 (48)	73 (47)	0·89
Bacteremia	71 (14)	25 (16)	0·48
Multilobar infiltration	231 (45)	65 (42)	0·50
Pleural effusion	69 (14)	39 (26)	**0·001**
Acute renal failure	290 (57)	67 (44)	**0·004**
Septic shock	140 (28)	58 (38)	**0·015**

Results are given as n (%) or mean±SD. Percentages calculated on non-missing data.

APACHE = acute physiology and chronic health evaluation; CURB-65 = confusion, elevated blood urea nitrogen, respiratory rate and blood pressure plus age ≥65 years; IMV = invasive mechanical ventilation; PSI = pneumonia severity index; SD = standard deviation.

### Microbiologic findings

An etiologic diagnosis of pneumonia was established in 336 (51%) patients. The rate of etiologic diagnosis and polymicrobial etiology was higher in patients from the IMV group (Table 4). *Streptococcus pneumoniae* was the main pathogen and did not differ between both groups. *Legionella pneumophila* was less frequent in patients from the IMV group.

**Table 4:** Microbial etiology of the study population.

Pathogen	No IMV n = 510	IMV n = 154	p-value
Patients with defined etiology	245 (48)	91 (59)	**0·016**
*Streptococcus pneumoniae*	135 (55)	51 (56)	0·98
with bacteremia	52 (10)	18 (12)	0·76
*Legionella pneumophila*	23 (9)	2 (2)	**0·046**
Respiratory viruses	34 (14)	19 (21)	0·16
Atypical bacteria	17 (7)	3 (3)	0·32
*Chlamydophila pneumoniae*	6 (2)	1 (1)	0·73
*Mycoplasma pnemoniae*	6 (2)	2 (2)	0·79
*Coxiella burnetti*	6 (2)	0 (0)	0·30
*Staphylococcus aureus*	12 (5)	8 (9)	0·27
*Pseudomonas aeruginosa*	18 (7)	7 (8)	0·90
*Haemophilus influenzae*	8 (3)	8 (9)	0·068
*Escherichia coli*	10 (4)	3 (3)	0·99
Other *Streptococcus* species	3 (1)	2 (2)	0·88
*Klebsiella pneumoniae*	3 (1)	0 (0)	0·68
*Moraxella catarrhalis*	3 (1)	1 (1)	0·64
Other microorganisms	13 (5)	8 (9)	---
Polymicrobial	35 (14)	22 (24)	**0·047**

Results are given as n (%). Percentages calculated on non-missing data. The percentages of pathogens are related to the number of patients with etiologic diagnosis in each group, except for bacteremia due to *Streptococcus pneumoniae*, which is calculated related to the total number of patients in each group.

IMV = invasive mechanical ventilation.

### Length of stay and outcome variables

The overall 30-day mortality rate was 145 (22%). The ICU and hospital stay were longer, and the 30-day mortality higher, in patients from the IMV group (Table 5).

**Table 5:** Site of admission, length of stay, treatment adequacy and outcome variables.

Variable	No IMV n = 510	IMV n = 154	p-value
ICU admission *	210 (41)	153 (99) ^†^	**<0·001**
ICU stay (days) *^‡^	4 (3;7)	10 (6;19)	**<0·001**
Hospital stay (days)	10 (7;14)	20 (12;33)	**<0·001**
Adequate empiric treatment ^§^	199 (92)	75 (89)	0·51
30-day mortality	94 (18)	51 (33)	**<0·001**

Results are given as n (%) or median (interquartile range). Percentages are calculated on non-missing data.

* Intermediate care units are also included.

^†^ The patient of the IMV group not admitted to ICU was extubated in the emergency room.

^‡^ Data calculated for patients admitted to an ICU only.

^§^ Data calculated for patients with defined bacterial etiology only.

ICU = intensive care unit; IMV = invasive mechanical ventilation; NIV = non-invasive ventilation; SD = standard deviation.

Among different variables associated with 30-day mortality in the univariate analysis (Table 6), IMV was independently associated with increased 30-day mortality in the multivariate analysis, together with septic shock, lower PaO_2_/FiO_2_ ratio, and higher levels of serum K^+^. The area under the ROC curve of the model to predict 30-day mortality was 0·78 (95% CI 0·70 to 0·86).

**Table 6:** Significant univariate and multivariate weighted logistic regression analyses for the prediction of 30-day mortality.

Variable	Univariate			Multivariate *		
	OR	95% CI	p-value	OR	95% CI	p-value
Age (+10 yrs.)	1·35	1·17 to 1·54	<0·001	-	-	-
Tobacco consumption				-	-	-
No	1	-	-	-	-	-
Former	0·76	0·50 to 1·15	0·19	-	-	-
Current	0·48	0·28 to 0·81	0·006	-	-	-
Alcohol abuse						
No	1	-	-	-	-	-
Former	0·29	0·09 to 0·97	0·044	-	-	-
Current	0·74	0·45 to 1·24	0·25	-	-	-
APACHE-II at admission	1·05	1·01 to 1·10	0·028	-	-	-
Chronic cardiovascular disease	1·77	1·17 to 2·66	0·006	-	-	-
Chronic liver disease	1·93	0·96 to 3·87	0·061	-	-	-
Chronic neurologic disease	2·77	1·86 to 4·13	<0·001	-	-	-
Mental confusion	1·62	1·12 to 2·35	0·011	-	-	-
Shock	1·74	1·18 to 2·55	0·005	3·40	1·38 to 8·36	0·008
PSI risk classes IV-V	3·05	1·59 to 5·86	<0·001	-	-	-
CURB-65 score 3–5	1·86	1·25 to 2·77	0·002	-	-	-
Serum Creatinine (+1 mg/dL)	1·21	1·03–1·43	0·023	-	-	-
Platelets (+100 x 10^9^ cell/L)	1·22	1·02 to 1·47	0·034	-	-	-
PaO_2_/FiO_2_ (+10 mmHg)	0.97	0.95 to 1.00	0.079	0·92	0·86 to 0·98	0·011
Serum Na^+^ (+1 mEq/L)	1·03	1·00 to 1·06	0·044	-	-	-
Serum K^+^ (+1 mEq/L)	1·36	1·07–1·73	0·012	2·54	1·32 to 4·90	0·005
Arterial pH (+0.1 units)	0·78	0·66 to 0·93	0·006			
Invasive mechanical ventilation	2·18	1·44–3·31	<0·001	3·54	1·45 to 8·67	0·006

* Summary statistics of the multivariate model: Pearson chi-square test, value / df = 0·93; AIC value = 144·51.

AIC = Akaike's information criterion; APACHE = acute physiology and chronic health evaluation; CI = confidence interval; CURB-65 = confusion, elevated blood urea nitrogen, respiratory rate and blood pressure plus age ≥65 years; df = degrees of freedom; OR = odds ratio; PSI = pneumonia severity index.

The actual mortality of the IMV group was higher than that predicted by the APACHE-II score (33% *vs*. 26%, respectively). In contrast, the actual mortality of patients without IMV was lower than that predicted by this score (18% *vs*. 23.5%, respectively).

Among SCAP patients we distinguished those who met the major criteria from those who met the minor criteria only, according to IDSA/ATS 2007 definition. The mortality of patients with at least one major severity criteria was higher than that of patients with minor criteria only (86, 29% *vs*. 59, 16%, p<0.001). The actual mortality of patients with septic shock and those with IMV alone was higher than that predicted by the APACHE-II score, while for patients without major severity criteria, the actual mortality was lower than that predicted by this score (Table 7). Mortality was highest in patients with both septic shock and IMV.

**Table 7:** Mortality, severity at admission and length of stay for patients with and without major severity criteria.

Variable	No shock or IMV n = 370	Shock alone n = 140	IMV alone n = 96	IMV and shock n = 58	p-value
30-day mortality	59 (16)	35 (25)	29 (30)	22 (38)	**<0·001**
APACHE-II at admission	16±6	15±5	15±5	19±5	**<0·001**
Mortality predicted by APACHE-II	23·5%	21%	21%	32%	---
ICU stay (days) *^†^	5 (3;7)	3 (2;6)	10 (5;19)	11 (7;20)	**<0·001**
Hospital stay (days)	10 (7;14)	9 (6;16)	18 (12;32)	21 (13;35)	**<0·001**

Data are n (%), mean±SD or median (interquartile range). Percentages are calculated on non-missing data.

* Intermediate care units are also included.

^†^ Data calculated for patients admitted to an ICU only. APACHE = acute physiology and chronic health evaluation; ICU = intensive care unit; IMV = invasive mechanical ventilation.

The overall rate of ICU admission was 363 (55%), and was higher in patients with IMV (Table 5). In patients without IMV, those admitted to the ICU had lower 30-day mortality than those no admitted to the ICU (Table 8). Regarding severity characteristics of non-intubated patients, the ICU patients had more frequently septic shock, bacteremia, PaO_2_/FiO_2_ ≤250, and multilobar radiologic infiltrates than non-ICU patients. In contrast, non-ICU patients were older, had more frequently confusion/disorientation, acute and chronic renal failure, and chronic cardiovascular and neurological disease, and higher APACHE-II score and PSI and CURB-65 risk classes. After adjustment for potential confounders, ICU admission in non-intubated patients was not significantly associated with lower 30-day mortality (adjusted OR 0·77, 95% CI 0·36 to 1·62, p = 0·49).

**Table 8:** Characteristics of patients not subjected to invasive mechanical ventilation divided into those admitted and those not admitted to the intensive care unit.

Variable	Non-ICU patients n = 300	ICU patients n = 210	p-value
Age (years)	77±14	66±17	**<0·001**
Sex (male)	202 (67)	135 (64)	0·47
**Co-morbidities**:			
Chronic respiratory disease *	121 (40)	96 (46)	0·23
Chronic cardiovascular disease ^±^	81 (27)	35 (17)	**0·006**
Diabetes mellitus	71 (25)	45 (22)	0·46
Chronic neurological disease	98 (33)	27 (13)	**<0·001**
Chronic renal disease	48 (16)	14 (7)	**0·001**
Chronic liver disease	13 (4)	13 (6)	0·36
**Major and minor severity criteria**: [1]			
Septic shock	71 (24)	69 (33)	**0·022**
PaO_2_/FiO_2_ ≤250 *	164 (55)	140 (67)	**0·007**
Respiratory rate ≥30 breaths/min ^‡^	175 (58)	125 (60)	0·79
Creatinine level >1·5 mg/dL	188 (63)	102 (49)	**0·002**
Confusion/disorientation	159 (53)	86 (41)	**0·007**
Multilobar radiologic infiltrates	125 (42)	106 (51)	**0·049**
Hypotension (not meeting septic shock criteria)	42 (14)	41 (20)	0·096
Core temperature <36°C	35 (12)	23 (11)	0·80
White blood cell counts <4,000 cells/mm^3^	15 (5)	18 (9)	0·11
Platelet count <100,000 cells/mm^3^	12 (4)	5 (2)	0·32
**Other severity variables at hospital admission**:			
APACHE-II	16±5	15±5	**0·036**
PSI risk class IV-V	261 (87)	163 (78)	**0·005**
CURB-65 risk score 3–5	209 (70)	113 (54)	**<0·001**
Bacteremia	33 (11)	38 (18)	**0·023**
Hospital stay (days)	8 (6;12)	12 (9;16)	**<0·001**
Adequate empiric treatment ^§^	95 (93)	104 (90)	0·47
30-day mortality	70 (23)	24 (11)	**0·001**

Data are n (%), mean±SD or median (interquartile range). Percentages calculated on non-missing data.

* Chronic respiratory disease includes chronic obstructive pulmonary disease, asthma, bronchiectasis, and *sequelae* of pulmonary tuberculosis.

^±^ Chronic cardiovascular disease includes coronary artery disease, hypertensive or valvular heart diseases, and dilated myocardial disease of any cause.

^‡^ The use of non-invasive mechanical ventilation can substitute for respiratory rate ≥30 breaths/min or PaO_2_/FiO_2_ ≤250 [1].

^§^ Data calculated for patients with defined bacterial etiology only.

IMV = invasive mechanical ventilation; SD = standard deviation. APACHE = Acute Physiology And Chronic Health Evaluation; PSI = pneumonia severity index; CURB-65 = Confusion, elevated blood Urea nitrogen, Respiratory rate and Blood pressure plus age ≥65 years.

## Discussion

We studied patients with SCAP independently from the site of care, with particular emphasis on the use of IMV. The main findings of this study are: 1) patients invasively ventilated had a high 30-day mortality rate, 33%; and 2) IMV, together with septic shock, worse hypoxemia and increased serum potassium, was independently associated with increased mortality.

Despite recent advances, pneumonia remains the main cause of death from infection in developed countries [22]. Several studies have identified that patients with respiratory failure and IMV, severe sepsis/septic shock, and decompensated co-morbidities are at greater risk of death [2,6,23–25].

The use of IMV is a major determinant for ICU admission in patients with CAP [1,3]. Between 37% and 60% patients with CAP in the ICU may require IMV [3,26–28]. The mortality rates of ICU patients with CAP ranged between 13% and 28%, depending on the different series and whether ICU or hospital mortality was reported. Although IMV was significantly associated with increased ICU mortality in patients with SCAP [26,27], a multivariate analysis found that IMV was not an independent prognostic factor among these ICU patients [26].

Several studies have assessed the outcomes of patients with CAP that require IMV [29–32]. These studies were retrospective or, in one case, prospective historic data were analyzed [30], and included a limited number of patients, ranging between 85 and 124. The mortality rate of these ventilated patients was high, 32% and 55% for ICU mortality [29,32], and 46% and 56% for hospital mortality [30,31]. Even in patients with CAP treated with NIV, the hospital mortality of those intubated after NIV failure may be as high as 54% [33]. As expected, older age, co-morbidities, and higher severity indices of pneumonia and organ system dysfunction at admission were independently associated with mortality in these reports. These studies, however, did not assess whether the use of IMV was simply a marker of more acute severe disease or was a determinant of poor outcome.

To our knowledge, the present study has assessed for the first time the characteristics of a large, prospective and consecutive series of hospitalized patients with SCAP with special focus in the association of IMV with mortality. Compared to non-intubated patients, those who received IMV did not present higher severity scores at hospital admission according to APACHE-II, PSI or CURB-65 scores. However, the use of IMV independently predicted 30-day mortality. The contribution of IMV to mortality is reinforced by the finding that the actual mortality of these patients was higher than that predicted by the APACHE-II score. In contrast, the actual mortality of non-intubated patients was lower than that predicted by this score. Whatever the cause is, the use of IMV seems to give a surplus of mortality in this subgroup of SCAP patients. Based on these results, PSI, CURB-65, or APACHE-II scores were less suitable than IMV for a reliable identification of SCAP patients at higher risk for mortality in our population.

Septic shock was also an independent predictor of mortality in patients with SCAP. This is not surprising considering that shock is an accepted major severity criterion of CAP and that it is associated with clinical failure [34].

We think that the strong and independent association of both major criteria with mortality, particularly IMV or the combination of both, would serve in the selection of very severe populations for future trials that would test new antibiotics or co-adjuvant therapies for SCAP [35].

The majority of patients with SCAP did not require intubation and IMV. The higher CURB-65 in non-intubated patients reflects an older population with more frequent acute renal failure at admission, two major components of this score. Patients without IMV were also characterized by a lower rate of defined microbial etiology; this is not surprising if we consider that lower respiratory tract samples are easily obtained in intubated patients.

Non-intubated patients were admitted to the ICU preferentially for septic shock, worse hypoxemia or multilobar involvement. The more severe presentation of pneumonia did not result in a higher mortality in this group compared to non-intubated patients admitted to a general ward. This is probably due to a proper monitoring and treating in the ICU-group, as well as to the older age, and the more frequent cardiovascular and neurological diseases in the non-ICU group; all these variables were independent predictors for mortality in this population, regardless the severity of pneumonia presentation. This may explain why ICU admission of non-intubated patients with SCAP was not associated with different mortality when potential confounders were considered.

In our opinion, the most important strengths of this study are the large number of patients recruited, the prospective and consecutive collection of data, the focus on intubated and non-intubated patients, and the statistical analysis for the prediction of mortality, with the IPTWs used to account for biases due to observed confounders and the propensity score. There are, however, some limitations to be addressed. First, the long period of recruitment, 12 years, since the care of patients could have evolved during this time. However, our protocol for managing CAP did not change substantially during these years. Second, this study was conducted in a single centre and therefore the extrapolation of these findings to other settings must be done cautiously. Third, complete information on the type, number and duration of previous antibiotic treatment was not collected.

In conclusion, IMV independently predicted 30-day mortality in patients with SCAP. Patients invasively ventilated should be considered a different population with higher mortality for future clinical trials on new interventions addressed to improve mortality of SCAP.

## Supporting information

S1 File Database.sav. (SAV)
